# Surface-Based Functional Alterations in Early-Onset and Late-Onset Parkinson’s Disease: A Multi-Modal MRI Study

**DOI:** 10.3390/diagnostics13182969

**Published:** 2023-09-17

**Authors:** Min Wang, Changlian Tan, Qin Shen, Sainan Cai, Qinru Liu, Haiyan Liao

**Affiliations:** Department of Radiology, The Second Xiangya Hospital, Central South University, Changsha 410017, China; 208202085@csu.edu.cn (M.W.); tanchanglian@csu.edu.cn (C.T.); shenqin@csu.edu.cn (Q.S.); caisainanxyz@126.com (S.C.); liuqr1990@163.com (Q.L.)

**Keywords:** early-onset Parkinson’s disease (EOPD), late-onset Parkinson’s disease (LOPD), surface-based ReHo, premotor area, dorsolateral prefrontal lobe, somatosensory and motor area, dorsal visual area, multi-modal, magnetic resonance imaging (MRI), imaging biomarkers

## Abstract

This study used a surface-based method to investigate brain functional alteration patterns in early-onset Parkinson’s disease (EOPD) and late-onset Parkinson’s disease (LOPD) to provide more reliable imaging indicators for the assessment of the two subtypes. A total of 58 patients with Parkinson’s disease were divided into two groups according to age at onset: EOPD (≤50 years; 16 males and 15 females) and LOPD (>50 years; 17 males and 10 females) groups. Two control groups were recruited from the community: young adults (YC; ≤50 years; 8 males and 19 females) and older adults (OC; >50 years; 12 males and 10 females). No significant differences were observed between the EOPD and YC groups or the LOPD and OC groups in terms of age, sex, education, and MMSE scores (*p* > 0.05). No statistically significant differences were observed between the EOPD and LOPD groups in terms of education, H-Y scale, UPDRS score, or HAMD score (*p* > 0.05). Data preprocessing and surface-based regional homogeneity (2D-ReHo) calculations were subsequently performed using the MATLAB-based DPABIsurf software. The EOPD group showed decreased 2D-ReHo values in the left premotor area and right dorsal stream visual cortex, along with increased 2D-ReHo values in the left dorsolateral prefrontal cortex. In patients with LOPD, 2D-ReHo values were decreased in bilateral somatosensory and motor areas and the right paracentral lobular and mid-cingulate. The imaging characterization of surface-based regional changes may serve useful as monitoring indicators and will help to better understand the mechanisms underlying divergent clinical presentations.

## 1. Introduction

Parkinson’s disease (PD) is a common degenerative disease of the central nervous system, and the number of PD cases continues to increase [1]. Clinically, PD onset at ≤50 years is considered early-onset Parkinson’s disease (EOPD) (5–10% of PD cases) [2], whereas onset at >50 years is considered late-onset Parkinson’s disease (LOPD).

Compared to late-onset Parkinson’s disease (LOPD), early-onset Parkinson’s disease (EOPD) is easily ignored and misdiagnosed due to its heterogeneous clinical presentation and relatively atypical symptoms. The incidence of dystonia is higher in EOPD. Patients with EOPD experience fewer gait difficulties early in the disease course compared to patients with LOPD [3], whereas the incidence of gait disorders is more likely in LOPD. Depression and vision loss are the most common non-motor symptoms in EOPD [4]. However, non-motor symptoms are more frequent and severe in LOPD, including an earlier and higher incidence of hyperkinesia and symptom fluctuations during treatment. Despite the differences in features in EOPD and LOPD, the neural mechanisms underlying these differences remain unclear.

Neuroimaging studies with regular Computed Tomography (CT) and conventional magnetic resonance imaging (MRI) sequences showed no characteristic changes between patients with EOPD and LOPD. Hence, special sequences and MRI algorithms could effectively demonstrate specific structural differences in these two subtypes [5,6,7,8]. However, some studies have reported contradictory results. For example, Sheng et al. [5] found reduced ReHo values in the putamen of both the EOPD and LOPD groups, whereas Xuan et al. [6] observed reduced gray matter density in the putamen only in the EOPD group. Another study [7] reported reduced DC values only in the putamen of the LOPD group. Notably, these studies were voxel-based. The topology of the cerebral cortex resembles a highly folded two-dimensional slice, and some functionally distant brain structures are closely connected in a three-dimensional space (such as the brain gyrus near the cerebral sulcus). Therefore, traditional voxel-based MRI analysis may not accurately reflect the intrinsic lamellar organization of the cerebral cortex. In contrast, surface-based analysis combines tissue classification and deformable model segmentation to analyze the inner and outer surfaces of the cortex, followed by the vertices of the triangles as the basic units of the cortical surface using a triangular grid mosaic on the surface. Finally, further calculations are performed according to the vertices of the inner and outer surfaces. Surface-based analysis is superior to voxel-based analysis in segmentation, signal-to-noise ratio, and reproducibility of the algorithm [9].

To our knowledge, previous studies have mostly been single-modal, and the surface-based analysis has generally been structural analysis. Therefore, this study aimed to integrate structure and function to identify more specific neuroimaging biomarkers in patients with EOPD and LOPD. Among the various indices for functional brain imaging studies, ReHo refers to the similarity in BOLD signal changes of adjacent voxels in the same time series. An increase in ReHo indicates an increase in the consistency of neuronal activity in the local brain region, and it is widely used to provide information about local activity within a small region of the brain. Surface-based ReHo (2D-ReHo) is more precise for the intrinsic functional organization with high re-test reliability [10].

We hypothesized that EOPD and LOPD have different patterns of brain functional alteration. By using 2D-ReHo, some specific brain regions may show functional alterations in EOPD and LOPD compared to healthy controls with similar age and sex. These brain regions may be associated with different neuropathophysiological mechanisms in EOPD and LOPD.

## 2. Materials and Methods

### 2.1. Participants

The study protocol was approved by the Medical Ethics Committee of our institute. The patients were informed before enrollment to ensure voluntary participation in the study and to sign an informed consent document.

A total of 58 patients with PD were recruited from October 2015 to March 2022. The inclusion criteria were as follows: (1) they met the British Brain Bank PD diagnostic criteria; (2) they were right-handed; (3) drug-naïve PD; (4) no intelligence impairment as evaluated by MMSE [11]; and (5) they can finish the MRI examination. The exclusion criteria were as follows: (1) they had unsuitable or contraindicated MRI examinations; (2) they had claustrophobia; (3) they felt discomfort during the MRI examination and could not continue the examination to completion; (4) they had a history of long-term alcohol abuse or other histories of drug abuse; (5) failure to complete the clinical scale assessment; and (6) they had other neurological or psychiatric illnesses. In total, 58 patients with PD were finally qualified for enrollment in this study. They were divided into two groups according to age of onset: the EOPD group (≤50 years) and the LOPD group (>50 years).

A total of 49 healthy participants were recruited from the community for the control groups and classified into younger controls (YC) with ages similar age to that of the patients with EOPD (8 males and 19 females) and older controls (OC) with ages similar to those of the patients with LOPD (12 males and 10 females).

Before MRI scanning, all participants were evaluated and recorded by the same attending neurologist, with all volunteers completing the Mini Mental State Examination scale (MMSE) and the Hamilton depression scale (HAMD). Patients with PD also recorded their medical history and disease duration and completed the Unified Parkinson’s Disease Rating Scale (UPDRS) and the Hoehn–Yahr scale (H-Y). The demographic and clinical data of all the participants are presented in Table 1.

### 2.2. MR Data Acquisition and Preprocessing

All MRI data were obtained using the same 3.0T MRI scanner (MAGNETOM Skyra; Siemens Healthineers, Erlangen, Germany) with a 16-channel head coil. The scans were performed by the same qualified technician. The MRI scanning procedure was as follows: after completing the localization scan, conventional T2-weighted imaging and T2 FLAIR imaging were performed sequentially, followed by resting-state scanning. The parameters of the resting sequence were as follows: number of scanned layers: 39; layer thickness: 3.5 mm; repetition time TR: 2500 ms; echo time TE: 25 ms; voxel size: 3.8 × 3.8 × 3.5 mm; flip angle: 90°; field of view: 240 mm; acquisition matrix: 64 × 64 mm; whole brain volume: 200. The scanned images were stored in digital imaging and communication of medicine (DICOM) format on a disk.

For data preprocessing and cortical-based ReHo calculation, the MRI resting-state data-processing software DPABIsurf (DPABI_V6.1) [12], running on MatLab 2018b, were used. The main steps included: (1) converting the collected resting-state functional MRI data from DICOM to NIFTI format, (2) converting the NIFTI format data to Brain Imaging Data Structure (BIDS) format using DPABIsurf, (3) evoking through DPABIsurf fMRIprep to pre-process structural and functional MRI, including skull stripping, spatial normalization, brain tissue segmentation, T1-weighted image-based brain surface reconstructions and temporal layer correction of resting-state functional images, head motion correction (exclusion criteria: head movement > 0.5 mm or angular rotation > 0.5° in any direction), and spatial rearrangement, followed by projection onto the FreeSurfer fsaverage5 surface space, (4) surface-based ReHo calculation using DAPBIsurf after regression of covariates, (5) surface smoothing using DPABIsurf, (6) surface-based threshold-free cluster enhancement (TFCE) for multiple comparison correction [13,14], and (7) presentation of the final results using DPABISurf_VIEW.

### 2.3. Statistical Analysis

SPSS V22.0 software was used for statistical analysis; the normality of measurement data was examined using the Kolmogorov–Smirnov (K-S) test. Data with normal distribution were compared between groups using the two sample T-tests, whereas data with non-normal distribution were compared using the Mann–Whitney U test. Fisher exact test was used to test the gender difference. The statistical significance threshold was set at *p* < 0.05.

### 2.4. Surface-Based ReHo Calculation

Surface-based-ReHo was generated for each subject using DPABIsurf software, as previously described [12]. Two-sample *t*-tests, with gender as a covariate, were used to evaluate the surface-based ReHo differences in each group (EOPD vs. YC and LOPD vs. OC), and the significance threshold was set at *p* < 0.05 for each subject and *p* < 0.025 for each hemisphere (TFCE-corrected).

### 2.5. Correlation Analysis

The brain regions showing significant differences in surface-based ReHo, in PD and HC, were identified as regions of interest (ROI). Spearman correlations were calculated with the mean values of each ROI and multiple clinical scales, including UPDRS, UPDRS part3, MMSE, H-Y stage, and HAMD. *p* value < 0.05 was considered statistically significant.

## 3. Results

### 3.1. Demographics and Clinical Characteristics

No significant differences were observed between the EOPD and young adult control (YC) groups or the LOPD and older adult control (OC) groups in terms of age, sex, education, and MMSE scores (*p* > 0.05), except for the HAMD scores (*p* < 0.05).

No statistically significant differences were observed between the EOPD and LOPD groups in terms of education, H-Y scale, UPDRS score, or HAMD score (*p* > 0.05), except for age, age at disease onset, and MMSE score (*p* < 0.05; Table 1).

### 3.2. Differences in Surface-Based ReHo

**Figure 1:**
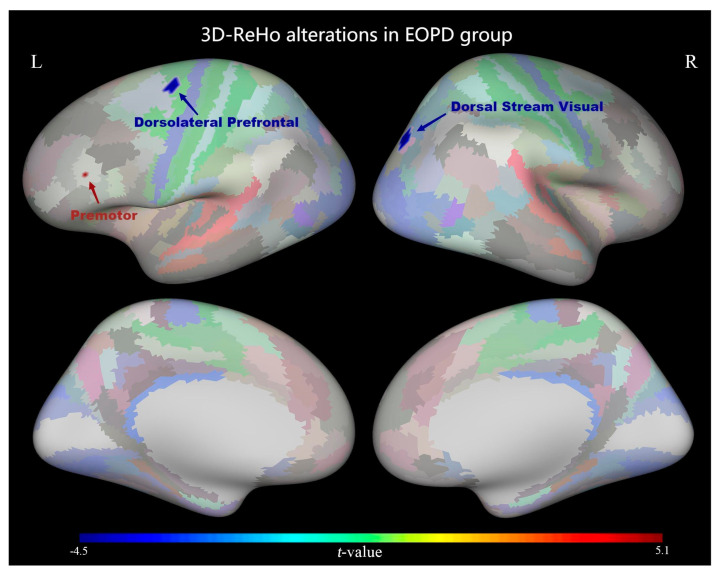
Surface−based regional homogeneity (ReHo) differences between the early−onset Parkinson’s disease (EOPD) and younger control (YC) groups. ReHo was decreased in the left premotor area and right dorsal stream visual cortex and increased in the left dorsolateral prefrontal cortex of the EOPD group (*p* < 0.05, TFCE−corrected).

Patients with EOPD showed decreased ReHo in the left premotor area and right dorsal stream visual cortex and increased ReHo values in the left dorsolateral prefrontal cortex (*p* < 0.05, TFCE corrected; Figure 1 and Table 2).

**Figure 2:**
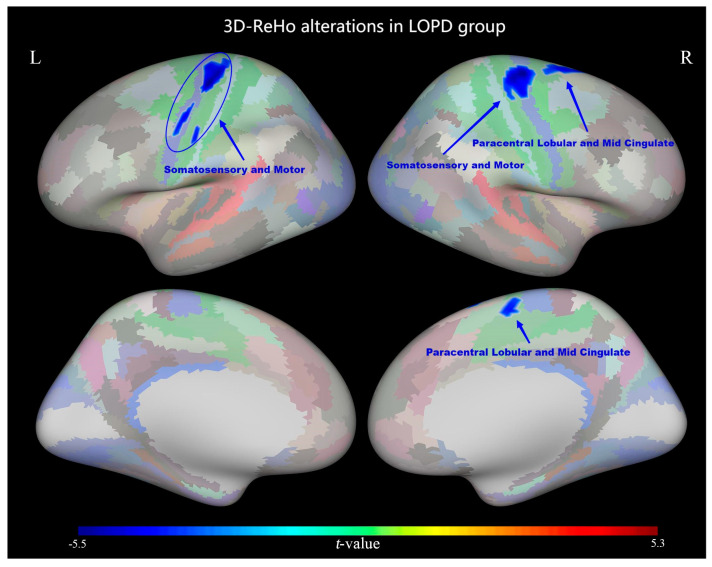
Surface−based regional homogeneity (ReHo) differences between the late−onset Parkinson’s disease (LOPD) and older control (OC) groups. ReHo was decreased in bilateral somatosensory and motor areas and the right paracentral lobular and mid−cingulate of the LOPD group (*p* < 0.05, TFCE−corrected).

However, patients with LOPD showed decreased ReHo values in the bilateral somatosensory and motor areas and the right paracentral lobular and mid-cingulate (*p* < 0.05, TFCE-corrected; Figure 2 and Table 3).

### 3.3. Correlation Analysis

**Figure 3:**
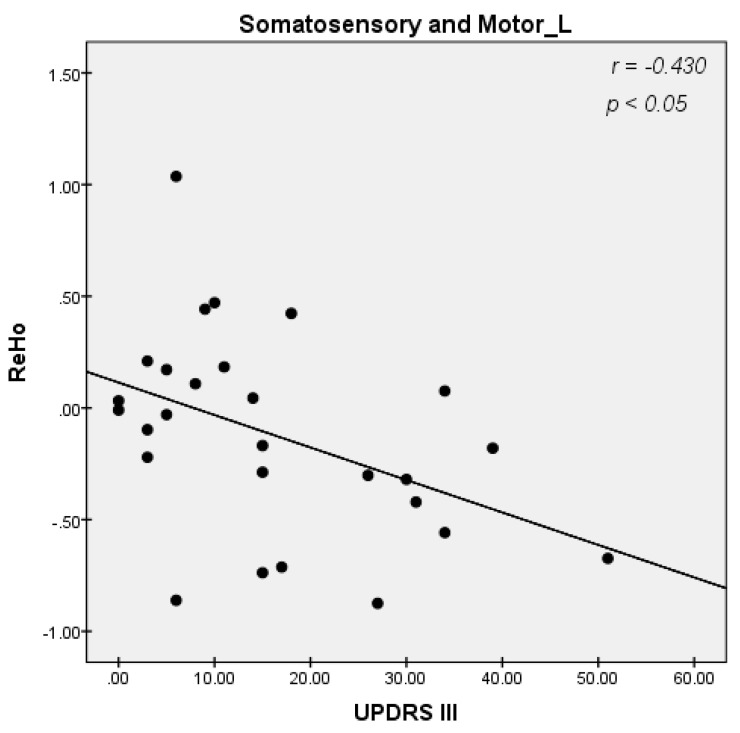
Correlations diagram between the mean value of regional homogeneity (ReHo) in the left somatosensory and motor area and the score of the third part of the Unified Parkinson’s Disease Rating Scale (UPDRS III) in patients with late−onset Parkinson’s Disease (LOPD) (r = − 0.430, *p* < 0.05, Bonferroni-corrected, Spearman correlation).

**Figure 4:**
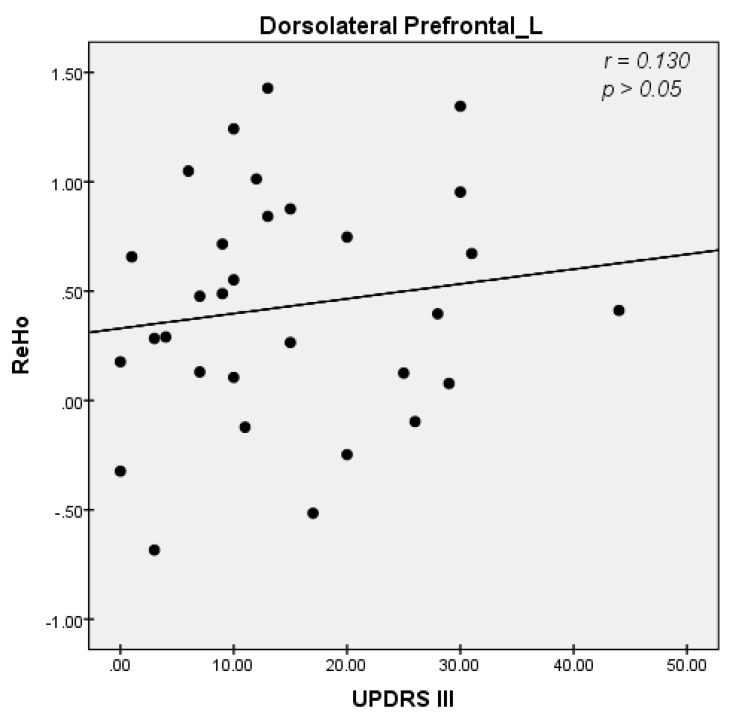
Correlation diagrams for the mean values of regional homogeneity (ReHo) in the left dorsolateral prefrontal cortex (DLPFC) and the score of the third part of the Unified Parkinson’s Disease Rating Scale (UPDRS III) in patients with early−onset Parkinson’s disease (EOPD) (r = 0.130, *p* > 0.05, uncorrected, Spearman correlation).

We explored ReHo alterations with multiple clinical scales, including the UPDRS score, the score of the third part of UPDRS, the MMSE score, the H-Y stage, and the HAMD score, to find correlations between clinical manifestations and ReHo alterations. However, our results showed no significant correlations except for a significant negative correlation between the mean value of ReHo in the left somatosensory and motor area with the score of the third part of UPDRS in patients with LOPD (r = −0.430, *p* = 0.025) (Figure 3). Interestingly, the ReHo value of DLPFC increased with the UPDRS score and the score of the third part of UPDRS in patients with EOPD, but the statistical correlation was not significant (r = 0.1300, *p* = 0.4840) (Figure 4).

## 4. Discussion

### 4.1. ReHo Value Alteration in Motor-Related Brain Regions Was Observed in Both EOPD and LOPD

Early-onset Parkinson’s disease (EOPD) and late-onset Parkinson’s disease (LOPD EOPD and LOPD) are two subtypes of PD that differ in risk factors, clinical features, and disease course. Bradykinesia is the main symptom of EOPD; however, gait problems are more common in patients with LOPD. Functional abnormalities in motor-related brain regions were observed in patients with EOPD and LOPD in this study, but the specific brain regions involved varied. The ReHo value decreased in the left premotor area in EOPD, whereas it decreased in the paracentral lobular and mid-cingulate and bilateral somatosensory and right motor areas in LOPD. Furthermore, the EOPD group showed decreased ReHo values in the visual cortex of the right dorsal stream visual cortex and increased ReHo values in the left dorsolateral prefrontal cortex. The decreased ReHo values represent a decrease in the consistency of neuronal activity in the brain regions, suggesting that there may be functional alterations in these regions.

Motor symptoms are mainly controlled by somatosensory and motor centers. The motor center comprises the supplementary motor area (SMA), the premotor area (PMA), and the primary motor area (M1), where the SMA and PMA are responsible for creating the motor program. The somatosensory system is divided into four sub-areas (areas 1, 2, 3a, and 3b) that receive muscle, skin, and joint afferents. The aforementioned cerebral cortex simultaneously sends information to the cerebellum, basal ganglia, brainstem, and spinal cord motor centers through the corticospinal and cortical brainstem tracts. To ensure precise movement, the cerebellum and basal ganglia are integrated into the cerebral cortex to modulate brain activity [15].

In this study, patients with EOPD had decreased ReHo values in the left premotor region, which is an essential component of the human brain’s motor area. This is a part of the development of autonomous motor programs. One hallmark of PD movement disorder is that automatic movement is more difficult than externally induced movement, which is consistent with impaired brain activity in the premotor region. Previous research on bradykinesia in PD has revealed that low activation of the premotor area may be the root cause of difficulties in preparing for autonomous movement, and dopamine-induced enhanced activation of the premotor area can accelerate the speed of exercise to some extent [16]. We suggest that the decrease in the ReHo value in the premotor region may be related to more frequent bradykinesia in EOPD.

### 4.2. Increased ReHo Values in the Dorsolateral Prefrontal Cortex May Be the Compensatory Mechanism Underlying Motor Symptoms in EOPD

Increased ReHo values have been observed in the dorsolateral prefrontal cortex (DLPFC) in patients with early-onset Parkinson’s disease (EOPD). Moreover, the ReHo value of DLPFC increased with the third part of UPDRS (Figure 4). An increase in the ReHo value indicates an increase in the consistency of neuronal activity in the brain regions. Based on our result, the increased ReHo value in DLPFC may be a compensatory mechanism underlying motor symptoms in patients with EOPD. The dorsolateral prefrontal cortex (DLPFC) is the core brain region of the central executive network, which is an important part of the basal ganglia-thalamo-cortical circuits (BGTC) and is tightly connected with multiple cortical and subcortical regions. The DLPFC is also involved in movement. For example, frozen gait is often accompanied by attention and executive dysfunction. Neuropsychological dual-task mode studies have found that performing cognitive tasks during walking activities induces freezing of gait and increasing cognitive load can aggravate freezing of gait [17]. Functional near-infrared spectroscopic studies have shown increased activity in the DLPFC when performing motor functions [18]. Furthermore, deep brain stimulation (DBS) studies have found that DLPFC function can be improved by DBS of the subthalamic nucleus (STN) [19]. Hyperdirect pathways in the prefrontal cortex and STN have been identified in primate studies, which can influence motor function [20]. Therefore, the dorsolateral prefrontal cortex not only modulates cognitive performance in patients with PD, but also has a significant impact on motor control. Previous electroencephalography (EEG) studies have also shown abnormalities in phase-amplitude coupling in the DLPFC in patients with PD [21]. The dorsolateral prefrontal cortex is thought to compensate for functional deficiencies in striatocortical networks, since previously complicated task-state functional studies of motor and cognitive dissociation have demonstrated the relative hyperactivation of this brain on the same side with more severe clinical symptoms during voluntary movement [22]. Furthermore, Transcranial direct current stimulation (tDCS) research shows that DLPFC tDCS exerted the most beneficial effects on dual-task walking and cortical modulation in participants with PD [23]. These findings are consistent with our results demonstrating increased DLPFC ReHo values in patients with EOPD but not in patients with LOPD. Notably, patients with LOPD were more likely to experience gait disturbances and more severe motor symptoms, which may be related to the absence of compensation in the DLPFC.

### 4.3. Decreased ReHo in the Visual Field of the Dorsal Stream May Be Related to Visual Loss Symptoms in EOPD

Our study also revealed decreased ReHo values in the dorsal stream visual of the early-onset Parkinson’s disease (EOPD) group. This is essential for transmitting visual information. Visual information is transmitted from the primary visual cortex along two pathways, dorsal and ventral, which are used to perform visual movement analysis, recognize object movement and self-movement, continuously detect the spatial position of stationary and moving objects, and accurately grasp the surrounding objects [24]. In this study, only patients with EOPD were shown to have lower ReHo values in the dorsal stream visual field, which may be connected to the fact that these patients were more likely to experience visual loss symptoms [4].

### 4.4. Decreased ReHo Values in LOPD at Bilaterally Sensorimotor Areas May Indicate That LOPD Patients Suffer from a More Widespread Impairment of Motor-Related Circuits

In this study, the ReHo values in the late-onset Parkinson’s disease (LOPD) group significantly decreased bilaterally in the sensorimotor areas. As previously mentioned, somatosensory areas process data primarily from the skin, muscles, and joint afferents. Direct or indirect projections of the sensory system interact with the brainstem, cerebellum, cortex, and subcortical regions of the brain. Thus, the choice of motor system and mode of movement is ultimately influenced by sensory stimulation. Previous EEG studies have also observed aberrant phase amplitudes in the somatomotor areas of patients with PD [25]. We propose that aberrant brain activity in the bilateral somatosensory motor areas of patients with LOPD may indicate a more pervasive impairment of motor-related circuits.

This study revealed the following: (1) premotor function was reduced in the LOPD group. Although the dorsolateral prefrontal lobe was improved and compensated for, patients with LOPD lacked functional augmentation that compensated for the dorsolateral prefrontal lobe. This lends credibility to the theory that Parkinson’s causes complicated damage to the motor cortex. (2) Different motor brain regions have suffered damage in patients with EOPD and LOPD, which may explain the differences in primary motor symptoms in these two subtypes. Bradykinesia is the primary initial symptom of EOPD, whereas gait disorders are more pervasive in patients with LOPD. (3) In patients with EOPD, the visual field of the ReHo values in the dorsal stream was also decreased, which may be related to the visual loss symptoms being more common in EOPD [4].

The investigation of surface-level changes in brain function in the EOPD and LOPD subtypes makes this a novel study. Unfortunately, owing to the cross-sectional study design, no longitudinal monitoring of brain imaging was performed. Hence, evaluating changes in these brain regions is difficult because of disease progression. Furthermore, the sample size was small. Future research will include the follow up of patients with PD, as well as a larger sample size. We carefully controlled variables like age, sex, and comorbidities in the control group. Nevertheless, it is imperative to acknowledge the presence of myriad factors capable of exerting influence upon 2D-ReHo values. Despite our diligent efforts to minimize their impact, it remains challenging to entirely eradicate all potential confounding influences. This is a limitation of the study.

## Figures and Tables

**Table 1:** Demographics and clinical details.

	EOPD	YC	LOPD	OC	*p* Value		
					EOPD vs. YC	LOPD vs. OC	EOPD vs. LOPD
Sample size (male/female)	16/15	8/19	17/10	12/10	0.113	0.574	0.434
Age (year) *	47 (45, 48)	49 (46, 50)	63 (58, 66)	61 (56, 65)	0.063	0.299	<0.001
Age of onset (year) *	44 (42, 46)	-	62 (55, 63)	-	-	-	<0.001
Duration of disease (month) *	12 (12, 36)	-	24 (12, 36)	-	-	-	0.630
Education (year) *	9 (5, 9)	9 (6, 11)	7 (3, 9)	7 (4, 12)	0.263	0.557	0.421
UPDRS *	25 (14, 42)	-	23 (14, 43)	-	-	-	0.516
UPDRS III *	12 (7, 25)	-	14 (5, 27)	-	-	-	0.981
H-Y scale *	1.5 (1.0, 2.5)	-	2 (1.0, 2.5)	-	-	-	0.398
HAMD *	5 (2, 10)	0 (0, 3)	8 (4, 10)	2.5 (0, 6)	<0.001	<0.001	0.487
MMSE *	28 (26, 29)	29 (26, 30)	25 (23, 27)	26.5 (23.75, 29)	0.086	0.585	0.011

* Median (interquartile range). Sex data were compared using Pearson’s chi-squared and Fisher exact test. Statistically significant differences were defined as *p* < 0.05. Abbreviations: EOPD, early-onset Parkinson’s disease; H-Y, Hoehn–Yahr; HAMD, Hamilton depression scale; LOPD, late-onset Parkinson’s disease; MMSE, Mini Mental State Examination; OC, older control; UPDRS, Unified Parkinson’s Disease Rating Scale; UPDRS III, the third part of the Unified Parkinson’s Disease Rating Scale; YC, younger control.

**Table 2:** Surface-based ReHo differences between EOPD and YC groups.

Brain Region (HCP)	Cluster Size	Peak T Value	Peak MNI Coordinate		
			X	Y	Z
Premotor L (54)	8	−4.289	54	55	96
Dorsolateral Prefrontal L (83)	1	4.692	−17	67	10
Dorsal Stream Visual R (16)	10	−5.004	−13	86	23

Abbreviations: EOPD, early-onset Parkinson’s disease; HCP, Human Connectome Project; MNI, Montreal Neurological Institute; YC, younger control.

**Table 3:** Surface-based ReHo differences between LOPD and OC groups.

Brain Region (HCP)	Cluster Size	Peak T Value	Peak MNI Coordinate		
			X	Y	Z
Somatosensory and Motor L (9)	43	−3.990	−30	−86	23
Somatosensory and Motor L (8)	39	−3.982	−19	8	44
Somatosensory and Motor L (53)	25	−4.654	−2	−8	63
Somatosensory and Motor R (8)	44	−5.538	−9	−2	67
Paracentral Lobular and Mid Cingulate R (55)	22	−3.940	−30	10	59
Somatosensory and Motor R (53)	19	−4.607	−8	−11	67
Paracentral Lobular and Mid Cingulate R (44)	19	−4.301	−19	24	65
Somatosensory and Motor R (9)	18	−4.135	−2	−11	63
Paracentral Lobular and Mid Cingulate R (43)	3	−3.884	−30	14	58
Paracentral Lobular and Mid Cingulate R (40)	2	−3.937	−30	12	57

Abbreviations: HCP, Human Connectome Project; LOPD, late-onset Parkinson’s disease; MNI, Montreal Neurological Institute; OC, older control.

## Data Availability

The raw data supporting the conclusions of this article will be made available by the authors, without undue reservation.
